# Supplementary material for: Assessment of the ecotoxicity of urban estuarine sediment using benthic and pelagic copepod bioassays
Source: PeerJ. 2018 May 30;6:e4936. doi: 10.7717/peerj.4936 (PMC5984583; doi:10.7717/peerj.4936)
Supplement: Supplemental Information 1 — Values are compared against the ANZECC ISQG-low and ISQG-High trigger values for sediments. Values have been normalized to 1% organic carbon. All values are reported in mg · kg−1 dry wt. ISQG = Interim Sediment Quality Guideline; ND = Not determined. [file peerj-06-4936-s001.docx]

|  | ANZECC  ISQG-Low | ANZECC ISQG-  High | Humber Drain | Humber Estuary | Old Tutaekuri Estuary | Old Tutaekuri Riverbed | Waitangi Estuary |
| --- | --- | --- | --- | --- | --- | --- | --- |
| 1-Methylnaphthalene | ND | ND | 0.0012 | <0.055 | <0.013 | <0.003 | <0.003 |
| 2-Methylnaphthalene | ND | ND | 0.0012 | <0.055 | <0.013 | <0.003 | <0.003 |
| Acenaphthene | 0.016 | 0.5 | 0.0036 | <0.055 | <0.013 | <0.003 | <0.003 |
| Acenaphthylene | 0.044 | 0.64 | 0.01 | <0.055 | <0.013 | <0.003 | <0.003 |
| Anthracene | 0.082 | 1.1 | 0.0244 | <0.055 | <0.013 | <0.003 | <0.003 |
| Benzo[a]anthracene | 0.261 | 1.6 | 0.144 | 0.008 | <0.013 | 0.015 | <0.003 |
| Benzo[a]pyrene (BAP) | 0.430 | 1.6 | 0.164 | 0.005 | <0.013 | 0.022 | <0.003 |
| Benzo[b]fluoranthene + Benzo[j]fluoranthene | ND | ND | 0.176 | 0.011 | <0.013 | 0.032 | <0.003 |
| Benzo[e]pyrene | 0.43 | 1.6 | 0.1 | <0.055 | <0.013 | 0.020 | <0.003 |
| Benzo[g,h,i]perylene | ND | ND | 0.1 | <0.055 | <0.013 | 0.021 | <0.003 |
| Benzo[k]fluoranthene | ND | ND | 0.0724 | <0.055 | <0.013 | 0.011 | <0.003 |
| Chrysene | 0.384 | 2.8 | 0.14 | <0.055 | <0.013 | 0.019 | <0.003 |
| Dibenzo[a,h]anthracene | 0.063 | 0.26 | 0.022 | <0.055 | <0.013 | <0.003 | <0.003 |
| Fluoranthene | 0. 6 | 5.1 | 0.288 | 0.008 | <0.013 | 0.034 | <0.003 |
| Fluorene | 0.019 | 0.54 | 0.0044 | <0.055 | <0.013 | <0.003 | <0.003 |
| Indeno(1,2,3-c,d)pyrene | ND | ND | 0.116 | <0.05 | <0.013 | 0.023 | <0.003 |
| Naphthalene | 0.16 | 2.1 | <0.0044 | <0.027 | <0.060 | <0.012 | <0.015 |
| Perylene | ND | ND | 0.0384 | <0.055 | <0.013 | 0.007 | <0.003 |
| Phenanthrene | 0.24 | 1.5 | 0.096 | <0.055 | <0.013 | 0.011 | <0.019 |
| Benzo[a]pyrene Potency Equivalency Factor (PEF) NES | ND | ND | 0.244 | <0.013 | <0.030 | 0.032 | <0.008 |
| Benzo[a]pyrene Toxic Equivalence (TEF) | ND | ND | 0.244 | <0.016 | <0.04 | 0.032 | <0.003 |
| Pyrene | 0.665 | 2.6 | 0.296 | 0.008 | <0.013 | 0.034 | 0.018 |
| Sum of low MW PAHs | 0.552 | 3.16 | 0.144 | 0.36 | <0.1 | 0.01 | <0.003 |
| Sum of high MW PAHs | 1.7 | 9.6 | 1.15 | 0.19 | 0.09 | 0.13 | <0.003 |
