# Supplementary material for: Assessment of the ecotoxicity of urban estuarine sediment using benthic and pelagic copepod bioassays
Source: PeerJ. 2018 May 30;6:e4936. doi: 10.7717/peerj.4936 (PMC5984583; doi:10.7717/peerj.4936)
Supplement: Supplemental Information 2 [file peerj-06-4936-s002.docx]

**Elutriate physicochemical properties from the five study sites.**

|  | pH | Total ammonia  (µg·L^-1^) | NPOC in (g·m^-3^) |
| --- | --- | --- | --- |
| Humber Drain | 8.03 | 1170 | 3.5 |
| Humber Estuary | 8.02 | 5 | 1.6 |
| Old Tutaekuri Riverbed | 8.02 | 960 | 3.8 |
| Old Tutaekuri Estuary | 8.02 | 220 | 1.5 |
| Waitangi Estuary | 8.06 | 760 | 2.5 |
