# Supplementary figures and images for: Assessment of the ecotoxicity of urban estuarine sediment using benthic and pelagic copepod bioassays
Source: PeerJ. 2018 May 30;6:e4936. doi: 10.7717/peerj.4936 (PMC5984583; doi:10.7717/peerj.4936)

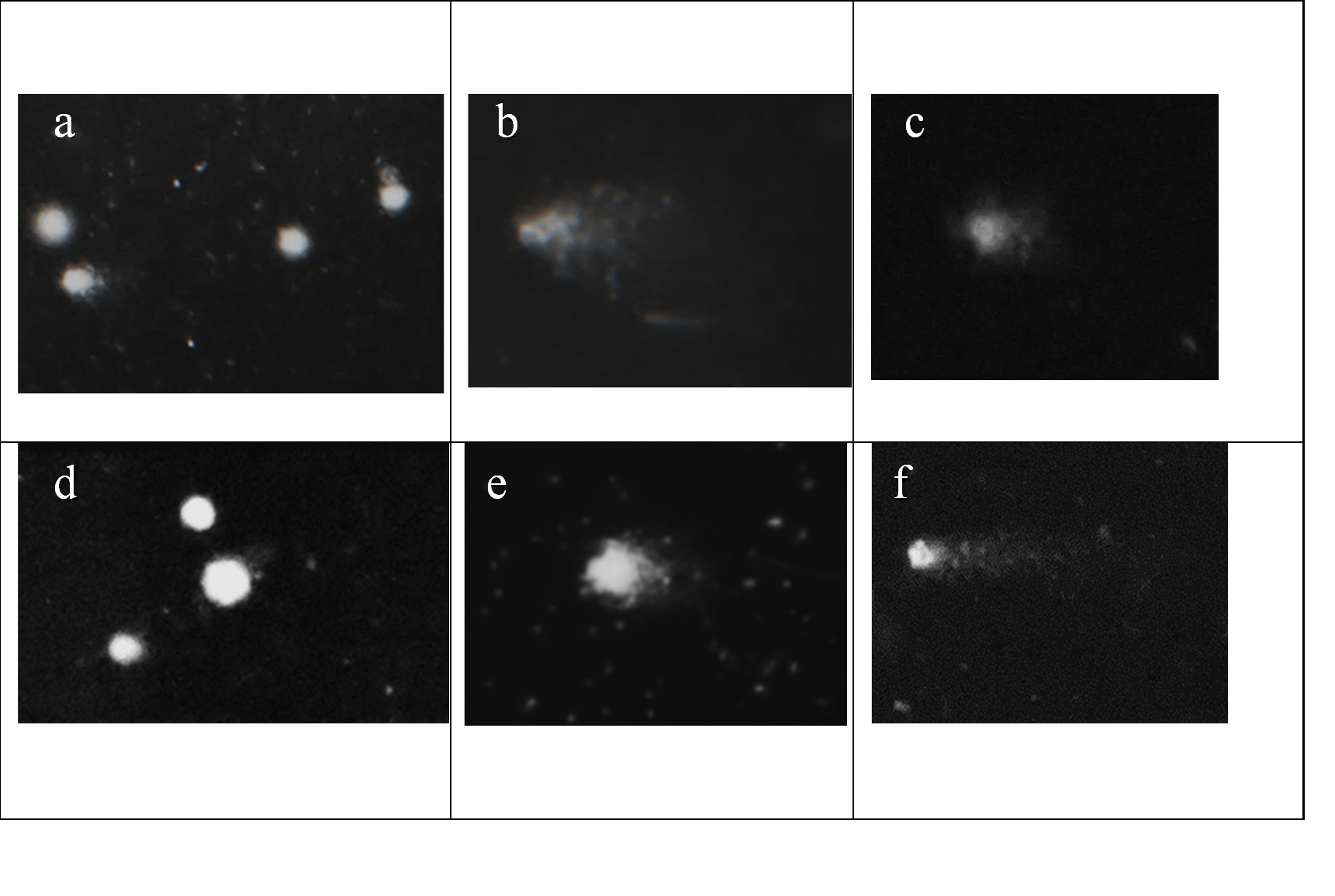

Supplement: Supplemental Information 3 — Comet assay images of copepod cell nucleoids exposed to different treatments: (a) Control, (b) UV exposure, (c) Humber Drain, (d) Old Tutaekuri Estuary site, (e) Humber Estuary site, (f) Old Tutaekuri Riverbed. [file peerj-06-4936-s003.png]

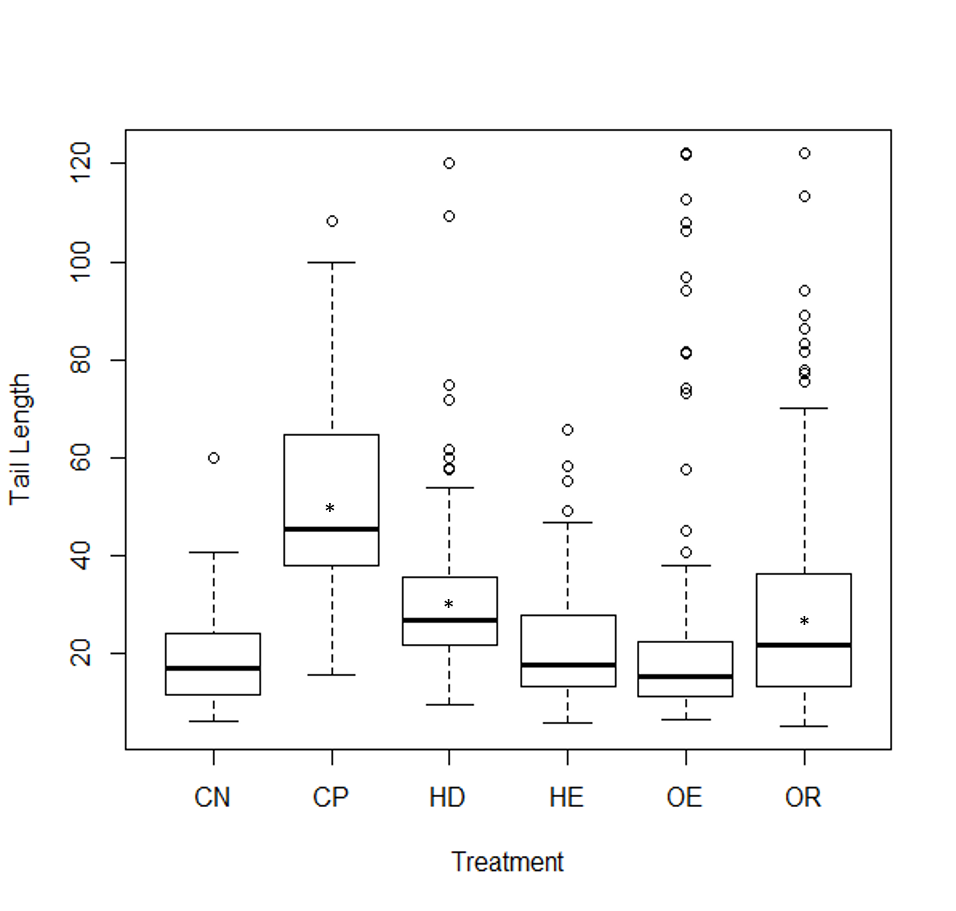

Supplement: Supplemental Information 4 — Negative control (CN), Positive control (CP), and elutriates from the Humber drain (HD), Humber Estuary (HE), Old Tutaekuri Estuary (OE) and Old Tutaekuri riverbed (OR)sediments. Humber drain (p < 0.01) Old Tutaekuri Riverbed (p < 0.001). [file peerj-06-4936-s004.png]
