# Supplementary material for: Assessment of the ecotoxicity of urban estuarine sediment using benthic and pelagic copepod bioassays
Source: PeerJ. 2018 May 30;6:e4936. doi: 10.7717/peerj.4936 (PMC5984583; doi:10.7717/peerj.4936)
Supplement: Supplemental Information 5 — Analytical results from elutriates prepared from the Humber Estuary, the Old Tutaekuri Estuary and Waitangi Estuary samples. All values are reported in mg · L−1. [file peerj-06-4936-s005.pdf]

Hill Laboratories
TRIED, TESTED AND TRUSTED

R J Hill Laboratories Limited
28 Duke Street Frankton 3204
Private Bag 3205
Hamilton 3240 New Zealand

T 0508 HILL LAB (44 555 22)
T +64 7 858 2000
E mail@hill-labs.co.nz
W www.hill-laboratories.com

ANALYSIS REPORT

SPv2

**Client:**	Cawthron Institute (Nelson)	**Lab No:**	1825352
**Contact:**	Maria Charry	**Date Received:**	15-Aug-2017
	C/- Cawthron Institute (Nelson)	**Date Reported:**	15-Sep-2017
	Private Bag 2	**Quote No:**	86159
	Nelson Mail Centre	**Order No:**	
	Nelson 7042	**Client Reference:**	
		**Submitted By:**	Maria Charry

Sample Type: Saline

**Sample Name:**		EHUM (8,14/8/17)	EWAI (8,11/8/17)	EOLD (8,11/8/17)	ELUHUM 08-Aug-2017 3:30 pm	ELUOLD 08-Aug-2017 4:00 pm
**Lab Number:**		1825352.4	1825352.5	1825352.6	1825352.7	1825352.8
Individual Tests						
Dissolved Arsenic*	g/m³	< 0.004	< 0.004	< 0.004	-	-
Dissolved Cadmium*	g/m³	< 0.0002	0.0005	< 0.0002	-	-
Dissolved Copper*	g/m³	< 0.0010	0.0083	< 0.0010	-	-
Dissolved Lead*	g/m³	0.0041	0.0040	0.0053	-	-
Dissolved Zinc*	g/m³	0.075	0.033	0.059	-	-
Total Ammoniacal-N	g/m³	-	-	-	1.17	0.96
Non-Purgeable Organic Carbon (NPOC)*	g/m³	3.5	2.5	3.8	-	-
Polycyclic Aromatic Hydrocarbons Trace in Water, By Liq/Liq						
Acenaphthene*	g/m³	< 0.000008	< 0.00002	< 0.000014	-	-
Acenaphthylene*	g/m³	< 0.000008	< 0.00002	< 0.000014	-	-
Anthracene*	g/m³	< 0.000008	< 0.00002	< 0.000014	-	-
Benzo[a]anthracene*	g/m³	0.000008	< 0.00002	< 0.000014	-	-
Benzo[a]pyrene (BAP)*	g/m³	0.000008	< 0.00002	< 0.000014	-	-
Benzo[b]fluoranthene + Benzo[j] fluoranthene*	g/m³	< 0.000008	< 0.00002	< 0.000014	-	-
Benzo[g,h,i]perylene*	g/m³	< 0.000008	< 0.00002	< 0.000014	-	-
Benzo[k]fluoranthene*	g/m³	< 0.000008	< 0.00002	< 0.000014	-	-
Chrysene*	g/m³	< 0.000008	< 0.00002	< 0.000014	-	-
Dibenzo[a,h]anthracene*	g/m³	< 0.000008	< 0.00002	< 0.000014	-	-
Fluoranthene*	g/m³	0.000009	< 0.00002	< 0.000014	-	-
Fluorene*	g/m³	< 0.000008	< 0.00002	< 0.000014	-	-
Indeno(1,2,3-c,d)pyrene*	g/m³	< 0.000008	< 0.00002	< 0.000014	-	-
Naphthalene*	g/m³	< 0.00004	< 0.00010	< 0.00007	-	-
Phenanthrene*	g/m³	< 0.000008	< 0.00002	< 0.000014	-	-
Pyrene*	g/m³	< 0.000008	< 0.00002	< 0.000014	-	-

**Sample Name:**		ELUWAI 08-Aug-2017 4:15 pm				
**Lab Number:**		1825352.9				
Individual Tests						
Total Ammoniacal-N	g/m³	0.76	-	-	-	-

Sample Type: Sediment

ilac-MRA IANZ ACCREDITED LABORATORY

This Laboratory is accredited by International Accreditation New Zealand (IANZ), which represents New Zealand in the International Laboratory Accreditation Cooperation (ILAC). Through the ILAC Mutual Recognition Arrangement (ILAC-MRA) this accreditation is internationally recognised.
The tests reported herein have been performed in accordance with the terms of accreditation, with the exception of tests marked *, which are not accredited.

Sample Type: Sediment						
	**Sample Name:**	SHUM 14-Aug-2017 1:00 pm	SWAI 14-Aug-2017 1:15 pm	SOLD 14-Aug-2017 1:10 pm		
	**Lab Number:**	1825352.1	1825352.2	1825352.3		
Individual Tests						
Dry Matter	g/100g as rcvd	79	56	76	-	-
Ammonium-N*	mg/kg dry wt	2.9	5.7	22	-	-
Total Organic Carbon*	g/100g dry wt	0.42	1.45	0.43	-	-

SUMMARY OF METHODS

The following table(s) gives a brief description of the methods used to conduct the analyses for this job. The detection limits given below are those attainable in a relatively clean matrix. Detection limits may be higher for individual samples should insufficient sample be available, or if the matrix requires that dilutions be performed during analysis.

Sample Type: Saline

Test	Method Description	Default Detection Limit	Sample No
Polycyclic Aromatic Hydrocarbons Trace in Water, By Liq/Liq*	Liquid / liquid extraction, SPE (if required), GC-MS SIM analysis [KBIs:4736,2695] Analysis performed at 1 Clyde Street, Hamilton	0.000005 g/m^3	4-6
Dissolved Arsenic*	Filtered sample, ICP-MS with dynamic reaction cell, ultratrace. Analysed at 1 Clyde Street, Hamilton. APHA 3125 B 22nd ed. 2012.	0.004 g/m^3	4-6
Dissolved Cadmium*	Filtered sample, ICP-MS, ultratrace level. Analysed at 1 Clyde Street, Hamilton. APHA 3125 B 22nd ed. 2012.	0.0002 g/m^3	4-6
Dissolved Copper*	Filtered sample, ICP-MS, ultratrace. Analysed at 1 Clyde Street, Hamilton. APHA 3125 B 22nd ed. 2012.	0.0010 g/m^3	4-6
Dissolved Lead*	Filtered sample, ICP-MS, ultratrace level. Analysed at 1 Clyde Street, Hamilton. APHA 3125 B 22nd ed. 2012.	0.0010 g/m^3	4-6
Dissolved Zinc*	Filtered sample, ICP-MS with dynamic reaction cell, ultratrace. Analysed at 1 Clyde Street, Hamilton. APHA 3125 B 22nd ed. 2012.	0.004 g/m^3	4-6
Total Ammoniacal-N	Saline sample. Phenol/hypochlorite colorimetry. Flow injection analyser. (NH4-N = NH4+-N + NH3-N). Analysis performed at 1 Clyde Street, Hamilton. APHA 4500-NH3 H 22nd ed. 2012.	0.005 g/m^3	7-9
Non-Purgeable Organic Carbon (NPOC)*	Acidification, purging to remove inorganic C, super-critical persulphate oxidation at 375°C, IR detection. Analysis performed at 1 Clyde Street, Hamilton. APHA 5310 C (modified) 22nd ed. 2012.	0.3 g/m^3	4-6

Sample Type: Sediment

Test	Method Description	Default Detection Limit	Sample No
Environmental Solids Sample Preparation	Air dried at 35°C and sieved, <2mm fraction. Used for sample preparation. May contain a residual moisture content of 2-5%. Analysis performed at 1 Clyde Street, Hamilton.	-	1-3
Dry Matter (Env)	Dried at 103°C for 4-22hr (removes 3-5% more water than air dry) , gravimetry. (Free water removed before analysis, non-soil objects such as sticks, leaves, grass and stones also removed). Analysis performed at 1 Clyde Street, Hamilton. US EPA 3550.	0.10 g/100g as rcvd	1-3
2M KCl Extraction*	2M potassium chloride extraction of as received fraction for analysis of NH4N, NO2N and NO3N. Analyst, 109, 549, (1984).	-	1-3
Ammonium-N*	2M potassium chloride extraction on as received fraction. Phenol/hypochlorite colorimetry. Discrete Analyser. APHA 4500-NH_3 F (modified) 22nd ed. 2012.	1.0 mg/kg dry wt	1-3
Total Organic Carbon*	Acid pretreatment to remove carbonates present followed by Catalytic Combustion (900°C, O2), separation, Thermal Conductivity Detector [Elementar Analyser]. Analysed at 1 Clyde Street, Hamilton.	0.05 g/100g dry wt	1-3

These samples were collected by yourselves (or your agent) and analysed as received at the laboratory.

Samples are held at the laboratory after reporting for a length of time depending on the preservation used and the stability of the analytes being tested. Once the storage period is completed the samples are discarded unless otherwise advised by the client.

This report must not be reproduced, except in full, without the written consent of the signatory.

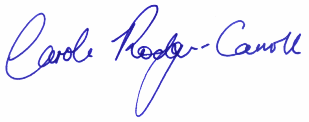

Carole Rodgers-Carroll BA, NZCS
Client Services Manager - Environmental
