# Supplementary material for: Assessment of the ecotoxicity of urban estuarine sediment using benthic and pelagic copepod bioassays
Source: PeerJ. 2018 May 30;6:e4936. doi: 10.7717/peerj.4936 (PMC5984583; doi:10.7717/peerj.4936)
Supplement: Supplemental Information 7 — Trace elements and polycyclic aromatic hydrocarbons analyzed for the Humber drain and the Old Tutaekuri riverbed samples. All values are reported in mg · L−1. [file peerj-06-4936-s007.pdf]

Hill Laboratories
TRIED, TESTED AND TRUSTED

R J Hill Laboratories Limited
28 Duke Street Frankton 3204
Private Bag 3205
Hamilton 3240 New Zealand

T 0508 HILL LAB (44 555 22)
T +64 7 858 2000
E mail@hill-labs.co.nz
W www.hill-laboratories.com

ANALYSIS REPORT

**Client:**	Cawthron Institute (Nelson)	**Lab No:**	1847686 SPv1
**Contact:**	Maria Charry	**Date Received:**	22-Sep-2017
	C/- Cawthron Institute (Nelson)	**Date Reported:**	05-Oct-2017
	Private Bag 2	**Quote No:**	86159
	Nelson Mail Centre	**Order No:**	
	Nelson 7042	**Client Reference:**	
		**Submitted By:**	Maria Charry

Sample Type: Saline

**Sample Name:**		Estold 19-Sep-2017	Esthum 19-Sep-2017	Hest 20-Sep-2017 2:00 pm	Oldest 20-Sep-2017 2:00 pm	
**Lab Number:**		1847686.1	1847686.2	1847686.3	1847686.4	
Individual Tests						
Dissolved Cadmium*	g/m^3	-	-	< 0.0002	< 0.0002	-
Dissolved Copper*	g/m^3	-	-	0.0010	0.0022	-
Dissolved Lead*	g/m^3	-	-	0.0016	0.0022	-
Dissolved Zinc*	g/m^3	-	-	0.033	0.044	-
Total Ammoniacal-N	g/m^3	-	-	0.005	0.22	-
Non-Purgeable Organic Carbon (NPOC)*	g/m^3	-	-	1.6	1.5	-
Polycyclic Aromatic Hydrocarbons Trace in Water, By Liq/Liq						
Acenaphthene*	g/m^3	< 0.000014	< 0.000014	-	-	-
Acenaphthylene*	g/m^3	< 0.000014	< 0.000014	-	-	-
Anthracene*	g/m^3	< 0.000014	< 0.000014	-	-	-
Benzo[a]anthracene*	g/m^3	< 0.000014	< 0.000014	-	-	-
Benzo[a]pyrene (BAP)*	g/m^3	< 0.000014	< 0.000014	-	-	-
Benzo[b]fluoranthene + Benzo[j] fluoranthene*	g/m^3	< 0.000014	< 0.000014	-	-	-
Benzo[g,h,i]perylene*	g/m^3	< 0.000014	< 0.000014	-	-	-
Benzo[k]fluoranthene*	g/m^3	< 0.000014	< 0.000014	-	-	-
Chrysene*	g/m^3	< 0.000014	< 0.000014	-	-	-
Dibenzo[a,h]anthracene*	g/m^3	< 0.000014	< 0.000014	-	-	-
Fluoranthene*	g/m^3	< 0.000014	< 0.000014	-	-	-
Fluorene*	g/m^3	< 0.000014	< 0.000014	-	-	-
Indeno(1,2,3-c,d)pyrene*	g/m^3	< 0.000014	< 0.000014	-	-	-
Naphthalene*	g/m^3	< 0.00007	< 0.00007	-	-	-
Phenanthrene*	g/m^3	< 0.000014	< 0.000014	-	-	-
Pyrene*	g/m^3	< 0.000014	< 0.000014	-	-	-

SUMMARY OF METHODS

The following table(s) gives a brief description of the methods used to conduct the analyses for this job. The detection limits given below are those attainable in a relatively clean matrix. Detection limits may be higher for individual samples should insufficient sample be available, or if the matrix requires that dilutions be performed during analysis.

Sample Type: Saline

Test	Method Description	Default Detection Limit	Sample No
Polycyclic Aromatic Hydrocarbons Trace in Water, By Liq/Liq*	Liquid / liquid extraction, SPE (if required), GC-MS SIM analysis [KBIs:4736,2695] Analysis performed at 1 Clyde Street, Hamilton	0.000005 g/m^3	1-2
Filtration, Unpreserved*	Sample filtration through 0.45µm membrane filter.	-	3-4
Filtration for dissolved metals analysis*	Sample filtration through 0.45µm membrane filter and preservation with nitric acid. APHA 3030 B 22nd ed. 2012.	-	3-4
Dissolved Cadmium*	Filtered sample, ICP-MS, ultratrace level. APHA 3125 B 22nd ed. 2012.	0.0002 g/m^3	3-4

ilac-MRA IANZ ACCREDITED LABORATORY

This Laboratory is accredited by International Accreditation New Zealand (IANZ), which represents New Zealand in the International Laboratory Accreditation Cooperation (ILAC). Through the ILAC Mutual Recognition Arrangement (ILAC-MRA) this accreditation is internationally recognised.
The tests reported herein have been performed in accordance with the terms of accreditation, with the exception of tests marked *, which are not accredited.

Sample Type: Saline			
**Test**	**Method Description**	**Default Detection Limit**	**Sample No**
Dissolved Copper*	Filtered sample, ICP-MS, ultratrace. APHA 3125 B 22nd ed. 2012.	0.0010 g/m^3	3-4
Dissolved Lead*	Filtered sample, ICP-MS, ultratrace level. APHA 3125 B 22nd ed. 2012.	0.0010 g/m^3	3-4
Dissolved Zinc*	Filtered sample, ICP-MS with dynamic reaction cell, ultratrace. APHA 3125 B 22nd ed. 2012.	0.004 g/m^3	3-4
Total Ammoniacal-N	Saline sample. Phenol/hypochlorite colorimetry. Flow injection analyser. (NH4-N = NH4+-N + NH3-N). APHA 4500-NH3 H 22nd ed. 2012.	0.005 g/m^3	3-4
Non-Purgeable Organic Carbon (NPOC)*	Acidification, purging to remove inorganic C, super-critical persulphate oxidation at 375°C, IR detection. APHA 5310 C (modified) 22nd ed. 2012.	0.3 g/m^3	3-4

These samples were collected by yourselves (or your agent) and analysed as received at the laboratory.

Samples are held at the laboratory after reporting for a length of time depending on the preservation used and the stability of the analytes being tested. Once the storage period is completed the samples are discarded unless otherwise advised by the client.

This report must not be reproduced, except in full, without the written consent of the signatory.

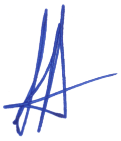

Ara Heron BSc (Tech)
Client Services Manager - Environmental
